# A new sequence logo plot to highlight enrichment and depletion

**DOI:** 10.1186/s12859-018-2489-3

**Published:** 2018-12-10

**Authors:** Kushal K. Dey, Dongyue Xie, Matthew Stephens

**Affiliations:** 1 Department of Statistics, University of Chicago, Chicago, 60637 USA; 2 Department of Human Genetics, university of Chicago, Chicago, 60637 USA

**Keywords:** Logo plots, Enrichment depletion, *EDLogo*, String symbols

## Abstract

**Background:**

Sequence logo plots have become a standard graphical tool for visualizing sequence motifs in DNA, RNA or protein sequences. However standard logo plots primarily highlight enrichment of symbols, and may fail to highlight interesting depletions. Current alternatives that try to highlight depletion often produce visually cluttered logos.

**Results:**

We introduce a new sequence logo plot, the *EDLogo* plot, that highlights both enrichment and depletion, while minimizing visual clutter. We provide an easy-to-use and highly customizable R package *Logolas* to produce a range of logo plots, including *EDLogo* plots. This software also allows elements in the logo plot to be strings of characters, rather than a single character, extending the range of applications beyond the usual DNA, RNA or protein sequences. And the software includes new Empirical Bayes methods to stabilize estimates of enrichment and depletion, and thus better highlight the most significant patterns in data. We illustrate our methods and software on applications to transcription factor binding site motifs, protein sequence alignments and cancer mutation signature profiles.

**Conclusions:**

Our new *EDLogo* plots and flexible software implementation can help data analysts visualize both enrichment and depletion of characters (DNA sequence bases, amino acids, etc.) across a wide range of applications.

**Electronic supplementary material:**

The online version of this article (10.1186/s12859-018-2489-3) contains supplementary material, which is available to authorized users.

## Background

Since their introduction in the early 1990s by Schneider and Stephens [1], sequence logo plots have become widely used for visualizing short conserved patterns known as *sequence motifs*, in multiple alignments of DNA, RNA and protein sequences. At each position in the alignment, the standard logo plot represents the relative frequency of each character (base, amino acid, etc.) by stacking characters on top of each other, with the height of each character proportional to its relative frequency. The characters are ordered by their relative frequency, and the total height of the stack is determined by the information content of the position. The visualization is so appealing that methods to produce logo plots are now implemented in many software packages (e.g. *seqLogo* [2], *RWebLogo* [3], *ggseqlogo* [4]) and web servers (e.g. *WebLogo* [5], *Seq2Logo* [6], *iceLogo* [7]).

Because the standard logo plot scales the height of each character proportional to its relative frequency, it tends to visually highlight characters that are *enriched*; that is, at higher than expected frequency. In many applications such enrichments may be the main features of interest, and the standard logo plot serves these applications well. However, sometimes it may be equally interesting to identify *depletions*: characters that occur *less often* than expected. One example of this, highlighted in [6], involves glycosylation: N-linked glycosylation sites in proteins are known to have the motif *N*-*X*- *S*/*T* where *X* is any amino acid apart from proline *P* [8, 9]. Another example involves the distribution of histone modifications across the genome: for example, Koch et al. [10] notes depletion of histone marks *H*4*AC* and *H*3*K*4*ME*1 at the gene start and gene end regions in lymphoblastoid cell lines. The standard logo plot represents strong depletion(s) by the *absence* of character(s), which can be difficult to discern visually.

To better highlight depletions in amino acid motifs, Thomsen et al. [6] suggests several alternatives to the standard logo plot. The key idea is to explicitly represent depletions using characters that occupy the negative part of the *y* axis. However, we have found that the resulting plots sometimes suffer from visual clutter – too many symbols, which distract from the main patterns of enrichment and depletion.

Here we suggest a simple solution to this problem, producing a new sequence logo plot – the *Enrichment Depletion Logo* or *EDLogo* plot – that highlights both enrichment and depletion, while minimizing visual clutter. In addition, we extend the applicability of logo plots to new settings by i) allowing each “character” in the plot to be an arbitrary alphanumeric string (potentially including user-defined symbols); and ii) allowing a different “alphabet” of permitted strings at each position. We also introduce Empirical Bayes statistical methods to stabilize estimates of enrichment and depletion, and thus better highlight the most significant patterns in data. All these new features are implemented in our R package, *Logolas*, which can produce generalized string-based logo and *EDLogo* plots. We illustrate the utility of the *EDLogo* plot and the flexibility of the string-based representation through several applications.

## Implementation

### Intuition

**Fig. 1:**
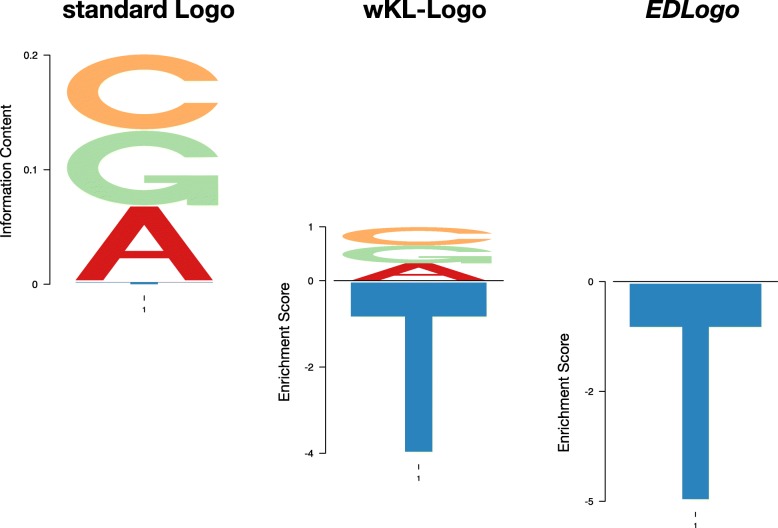
Illustration of the differences between standard logo, *EDLogo* and wKL-Logo representations. The figure shows how the different logos represent observed frequencies **p**=(*p*_*A*_,*p*_*C*_,*p*_*G*_,*p*_*T*_)=(0.33,0.33,0.33,0.01) (compared with a uniform background, **q**=(0.25,0.25,0.25,0.25)). The standard logo effectively represents **p** by highlighting that “A, C and G are enriched”; *EDLogo* represents it by highlighting “T is depleted”; wKL-Logo represents it as “A, C and G are enriched and T is depleted”. All are correct statements, but the *EDLogo* representation is the most parsimonious

In essence, the goal of a logo plot is to represent, at each position along the *x* axis, how a probability vector **p** compares with another probability vector **q**. For example, suppose that at a specific position in a set of aligned DNA sequences, we observe relative frequencies **p**=(*p*_*A*_,*p*_*C*_,*p*_*G*_,*p*_*T*_)=(0.33,0.33,0.33,0.01) of the four bases {*A*,*C*,*G*,*T*}. The goal of the logo plot might be to represent how **p** compares with the background frequencies of the four bases, which for simplicity we will assume in this example to be equal: **q**=(*q*_*A*_,*q*_*C*_,*q*_*G*_,*q*_*T*_)=(0.25,0.25,0.25,0.25). Verbally we could describe the change from **q** to **p** in several ways: we could say “*T* is depleted”, or “ *A*,*C* and *G* are enriched”, or “*T* is depleted, and *A*,*C* and *G* are enriched”. While all of these are valid statements, the first is the most succinct, and our *EDLogo* plot provides a visual version of that statement. The second statement is more in line with a standard logo representation, and the last is in essence the approach in [6] (also known as weighted Kullback Leibler Divergence Logo or wKL-Logo). See Fig. 1.

### The *EDLogo* plot

At a particular position, *j*, of a sequence (or other indexing set), let **p**=(*p*_1_,*p*_2_,…,*p*_*n*_) denote the probabilities of the *n* elements *C*_1_,…,*C*_*n*_ (which can be characters or strings) permitted at that position, and **q**=(*q*_1_,*q*_2_,…,*q*_*n*_) denote corresponding background probabilities. Define **r**=(*r*_1_,*r*_2_,…,*r*_*n*_) by:

1 $$  r_{i} = \tilde{r}_{i} - \text{median} \left (\left \{ \tilde{r}_{i} : i = 1, 2, \ldots, n \right \} \right),  $$

where

2 $$ \tilde{r}_{i}:= \log_{2} \frac{p_{i}}{q_{i}}.  $$

Then at position *j* along the *x* axis, the *EDLogo* plot plots the element *C*_*i*_, scaled to have height |*r*_*i*_|, and above the *x* axis if *r*_*i*_ is positive, or below the *x* axis if *r*_*i*_ is negative. Elements are stacked (from bottom to top) in order of increasing *r*_*i*_, so that the largest characters are furthest from the axis.

The basic strategy has close connections to ideas in [6], but with the crucial difference that we subtract the median in Eq. 1. As our examples will demonstrate, subtracting the median in this way – which can be motivated by a parsimony argument (see below) – can dramatically change the plot, and substantially reduce visual clutter.

Note that the *EDLogo* plot for **p** vs **q** is essentially a mirror (about the *x* axis) of the *EDLogo* plot for **q** vs **p** (e.g. Additional file 2: Figure S1). We call this the “mirror property”, and it can be interpreted as meaning that the plots treat enrichment and depletion symmetrically. This property is also satisfied by plots in [6], but not by the standard logo plot.

#### A model-based view

Suppose we model the relationship of **p** to **q** by

3 $$  p_{i} \propto \lambda_{i} q_{i}  $$

for some unknown (positive) “parameters” *λ*_*i*_. For example, this model would arise if **q** represents the underlying frequencies of elements in a population, and **p** represents the frequencies of the same elements in a (large) sample from that population, conditional on an event *E* (e.g. a transcription factor binding). Indeed, by Bayes theorem, under this assumption we would have

4 $$ p_{i} \propto \Pr(E| \text{element } {i}) q_{i}.  $$

Since the *p*_*i*_ must sum to 1, ${\sum }_{i} p_{i} = 1$, the model (3) implies

5 $$ p_{i} = \lambda_{i} q_{i}/ \sum\limits_{j} \lambda_{j} q_{j}.  $$

Now consider estimating the parameters **λ**. Even if **p** and **q** are observed without error, there is a non-identifiability in estimating **λ**: we can set *λ*_*i*_=*cp*_*i*_/*q*_*i*_ for any positive *c*. Equivalently, if we consider estimating the logarithms *l*_*i*_:= log*λ*_*i*_, we can set

6 $$  l_{i} = \log_{2}(p_{i}/q_{i}) + k  $$

for any constant *k*. Note that *r*_*i*_ in (1) has exactly this form, and so the vector **r** can be interpreted as an estimate of the vector ł. Furthermore, it is easy to show that, among all estimates of the form (6), **r** has the smallest sum of absolute values (see Additional file 1 for a rigorous proof). That is, **r** solves the optimization

7 $$  \mathbf{r} = \arg \min_{\text{\l}} \sum\limits_{i} |l_{i}|  $$

subject to the constraint (6).

Since the sum of absolute values of **r** is the total height of the stacked characters in the *EDLogo* plot, one can think of our choice of **r** as the *estimate of łthat produces the smallest stack of characters* – that is, the most “parsimonious” estimate.

### Interpretation

Roughly speaking, positive values of *r*_*i*_ can be interpreted as indicating characters that are “enriched” and negative values of *r*_*i*_ as indicating characters that are “depleted”. Formally we must add that here enrichment and depletion are to be interpreted as *relative to the median enrichment/depletion across characters*. This relative enrichment does not necessarily imply enrichment or depletion in some “absolute” sense: for example, *r*_*i*_ could be positive even if *p*_*i*_ is smaller than *q*_*i*_. For compositional data it seems natural that enrichment/depletion be interpreted relative to some “baseline”, and our choice of the median as the baseline is motivated above as providing the most parsimonious plot.

It may also help interpretation to note that for any two characters *i* and *i*^′^, the difference in their heights $\phantom {\dot {i}\!}r_{i}-r_{i^{\prime }}$ is equal to the log-odds ratio:

8 $$ r_{i}-r_{i^{\prime}} = \log_{2} \left(\frac{p_{i}/p_{i^{\prime}}}{q_{i}/q_{i^{\prime}}} \right).  $$

### Multiple solutions when *n* is even

When the number of classes *n* is even (*n*=4 DNA bases being a particularly relevant example) the definition of the median of $\tilde {r}_{1},\dots,\tilde {r}_{n}$, which is subtracted in (1) to minimize total stack height, is ambiguous. Conventionally, the median of an even number of observations is usually taken to be the mean of the two central observations. However, in terms of minimizing total stack height (optimization (7)), every real number between the two central observations (inclusive) performs equally well. For example, if $\tilde {r}=(0,0,+1,+1)$, subtracting the conventional median (0.5) yields *r*=(−0.5,−0.5,0.5,0.5) with total stack height 2, but subtracting any number between the two central observations (0 and 1) would lead to the same total stack height. Thus, if we measure parsimony by total stack height, there exist multiple equally-parsimonious plots, giving the user a decision to make.

Among these equally-parsimonious solutions, subtracting the smallest number corresponds to favoring an “enrichment” representation, whereas subtracting the largest number favors a “depletion” representation, and subtracting the median treads a middle ground between the two. See Additional file 2: Figure S6 for an illustration. None of these approaches is uniformly superior to another, but our sense is that – all other things being equal – users find an enrichment representation slightly more natural, and so we made this (subtracting the smallest number) the software default. One cost of this choice is that the plot no longer satisfies the mirror property; the mirror property is preserved by using the conventional median, which is a software option.

### Stabilizing estimates of $\tilde {r}_{i}$

The basic *EDLogo* plot described above typically works well provided that no probabilities *p*_*i*_ or *q*_*i*_ are very small (or zero!). Very small values of *p*_*i*_ or *q*_*i*_ can cause very large values of $|\tilde {r}_{i}|=|\log _{2} (p_{i}/q_{i})|$, and consequently large |*r*_*i*_| which can undesirably dominate the plot.

In practice we have found the most common source of this problem (unreasonably large |*r*_*i*_|) is unstable estimates of small probabilities from low counts of rare events. We found that the simplest solution to this problem – use of pseudocounts to stabilize estimates of small probabilities [11] – was only partially successful. We therefore developed a statistical approach that directly stabilizes estimates of $\tilde {\mathbf {r}}$ from count data. This approach uses Empirical Bayes shrinkage [12] to stabilize estimates of $\tilde {\mathbf {r}}$, and is especially effective in stabilizing estimates from low count events (see Additional file 1 for details). We produce an *EDLogo* plot from stabilized estimates for $\tilde {r}_{i}$ by plugging them into (1).

Although our stabilization method is ideally-suited to settings where **p** and **q** are estimated from count data, it can also be applied in other settings by supplying an “effective count” parameter that specifies the approximate precision of supplied values of **p** and **q**. (By default we assume an effective count of 1000, which means that **p** and **q** are precise to no more than 3 decimal places).

## Results

### Comparison with existing logo plots

**Fig. 2:**
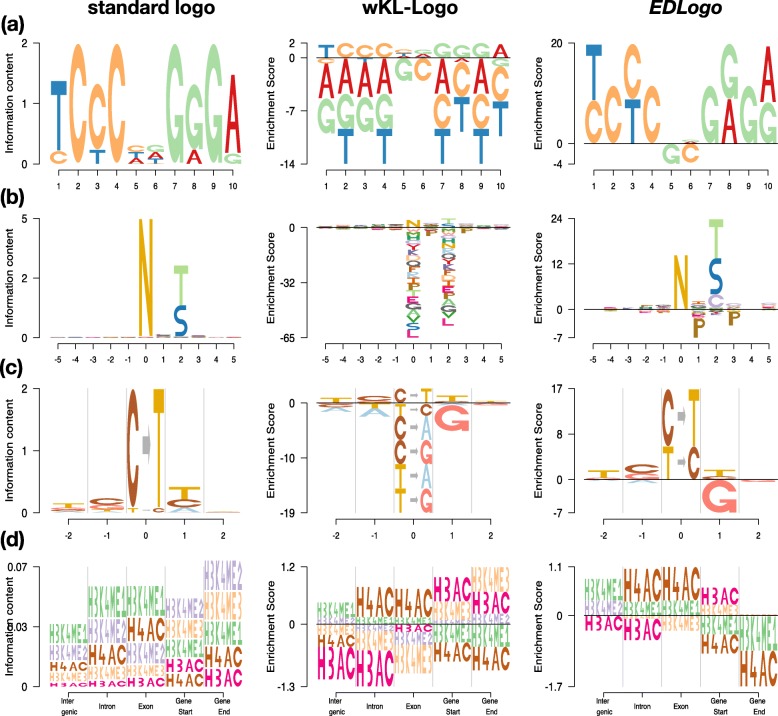
Comparison of standard logo plot, weighted KL (w-KL) logo plot and *EDLogo* plot on four examples. Panel (**a**): the transcription factor binding site of the EBF1-disc1 transcription factor from the ENCODE project [14]. Panel (**b**): The N-linked glycosylation motif, with the site of the *N* amino acid at the center (position 0), and 5 bases on either side of the *N* (based on data from UniprotKB [19]). The *EDLogo* clearly shows the *N*-*X*- *S*/*T* motif where *X* represents any amino acid except proline (*P*). Panel (**c**): a somatic mutation signature (signature 12 from [20]). The heights of the strings in the center of each plot (*C*→*G*, *C*→*T*, etc at position 0 on *x* axis) reflect the relative frequency of each substitution type among somatic mutations contributing to the signature, while the heights of the bases at flanking positions (-2,-1,1,2 on *x* axis) reflect the relative frequency of each base at positions flanking these somatic mutations. The depletion of G at position +1 - possibly occurring due to the rarity of CpG sites owing to de-amination of methylated cytosines - is clearest in the *EDLogo* plot. Panel (**d**): relative abundance of histone modification sites across various genomic regions in the lymphoblastoid cell line GM06990 (Table S2 in Koch et al. 2007 [10]). These examples illustrate the ability of the *EDLogo* plot to highlight both enrichment and depletion, while avoiding unnecessarily visual clutter. The last two examples also illustrate how our software allows arbitrary strings as elements in a logo plot

Figure 2 illustrates the *EDLogo* plot, and compares it with the standard logo and the weighted Kullback–Leibler logo (wKL-Logo) plot [6], in four diverse applications.

The first application (panel (a)) is a setting where the standard logo plot is widely used: visualizing transcription factor binding sites (TFBS) [13–18] (see Additional file 3: Table S1). Specifically, the plots represent the primary discovered motif *disc1* of Early B cell factor EBF1 from ENCODE [14]. This example showcases the effectiveness of the standard logo plot in highlighting enrichments: in our opinion it does this better than the other two plots, and in this sense the other plots should be viewed as complementing the standard plot rather than replacing it. This example also illustrates the difference between the wKL-Logo and *EDLogo* plots, both of which aim to highlight depletion as well as enrichment: the *EDLogo* plot introduces less distracting visual clutter than the wKL-Logo plot, producing a cleaner and more parsimonious visualization that better highlights the primary enrichments and depletions. In particular, the *EDLogo* plot is most effective at highlighting depletion of bases G and C at the two positions in the middle of the sequence. This depletion is hard to see in the standard logo because of its emphasis on enrichment, and less clear in the wKL-Logo due to visual clutter. This depletion pattern is likely meaningful, rather than a coincidence, since it was also observed in two other previously known motifs (*known3* and *known4*) of the same transcription factor [16, 18] (see Additional file 2: Figure S2).

The second example (panel (b)) shows an amino acid motif corresponding to *N*-linked glycosylation sites. These sites are expected to have the motif *N*-*X*- *S*/*T*, where *X* is any amino acid apart from proline *P* (data from UniProtKB [19]; see Additional file 3: Table S2). This was used by [6] as an example of a motif where depletion is an important biological feature. The depletion (of the *P* at position +1) is essentially impossible to see in the standard logo plot, is visually detectable in the wKL-Logo plot, and clearest in the *EDLogo* plot. Again, the *EDLogo* plot is more parsimonious than the wKL-Logo plot, and consequently the primary *N*-*X*- *S*/*T* motif stands out better in the *EDLogo* plot. In addition to showing depletion of *P* at the expected position (+1), the *EDLogo* plot also highlights depletion of *P* at position +3, suggesting an extended motif *N*-*X*- *S*/*T*-*X*.

The next two applications (panels (c) and (d) of Fig. 2) are non-standard settings that illustrate the use of general strings as “characters” in a logo plot, as well as providing further examples where the *EDLogo* plot is particularly effective at highlighting depletion as well as enrichment.

Panel (c) shows logo plots representing an estimated cancer mutation signature profile (signature 12) from a clustering analysis of a large number (nearly 70,000) somatic mutations by [20] (see Additional file 3: Table S3). Here we follow [20] in representing a mutational signature by the frequency of each mutation type (at position 0 on the *x* axis), together with base frequencies at the ± 2 flanking bases. We also follow the common convention of orienting the strand so that the mutation is from either a *C* or a *T*, yielding six possible mutation types: *C*→*T*, *C*→*A*, *C*→*G*, *T*→*A*, *T*→*C*, *T*→*G*. This Figure panel illustrates two important points. First, it illustrates the flexibility of our software package *Logolas*, which allows arbitrary strings in a logo. For all three logo plots (standard, wKL and ED) we use this to represent the six mutation types by six strings of the form *X*→*Y*, and we find the resulting plots easier to read than the *pmsignature* plots in [20] (see Additional file 2: Figure S3 for comparison). Additionally, it also shows that one can use different sets of permitted strings at different positions - strings are used to represent the mutation in the center, while characters are used to represent the flanking bases. Second, it illustrates a case where, in our opinion, the *EDLogo* plot is a better visual summary than the other plots. Specifically the *EDLogo* plot best highlights the primary aspects of this signature: enrichment of *C*→*T* mutations, and depletion of *G* at position +1. Here the depletion of *G* at +1 may be a bi-product of the enrichment of *C*→*T* mutations combined with the overall depletion of CpG sites in the genome due to deamination [21].

In this example the *EDLogo* plot and the standard logo plot differ on the enrichments they highlight at the central position: unlike the standard plot, the *EDLogo* plot highlights enrichment of *T*→*C* in addition to the primary enrichment *C*→*T*. This is due to an important difference between the plots: in *EDLogo* enrichments (and depletions) are plotted on a log scale, whereas in the standard plot they are on an absolute scale. This means that in the *EDLogo* plot it is *differences* in the heights of characters that matter (and can be interpreted as a log-odds-ratio; see Implementation), whereas in a standard plot it is *ratios* of heights. In this case the frequency of *C*→*T* is 0.96 and *T*→*C* is 0.03, with other mutations essentially absent. Consequently *T*→*Cis* enriched relative to other mutation types, but nowhere near as strongly as *C*→*T*. When these enrichments are plotted on the raw scale, as in the standard plot, essentially only the *C*→*T* enrichment is visible. On the log scale, both are visible. Which representation is preferable depends on how much one wants to emphasize subtler vs stronger enrichment patterns.

For readers interested in other cancer mutation signatures, we provide *EDLogo* plots for all 27 mutational signature profiles reported by [20] in Additional file 2: Figure S4.

Panel (d) shows logo plots summarizing the relative abundance of 5 different histone marks in different genomic contexts (data from lymphoblastoid cell line GM06990, Table S2 of [10], given in Additional file 3: Tables S4 and S5.) Relative abundances naturally yield compositional data that can be visualized in a logo plot. Again this example illustrates the potential to use strings in logo plots. It also represents an example where the *EDLogo* and wKL-Logo plots seem more informative than the standard logo plot. Specifically, the standard logo plot is dominated by the high deviation from background frequencies at the intergenic, exon and intron regions, and the differences in enrichments and depletions among regions are difficult to discern. In comparison, the *EDLogo* and wKL-Logo plots highlight a number of differences among regions (some of which are also noted in [10]). For example, both plots highlight the relative enrichment of H3AC and H3K4me3 near the start of genes, and corresponding relative depletion of H4AC and H3K4me1. Both plots also highlight relative enrichment of H3K4me1 compared with other marks in the intergenic, exonic and intronic regions; the relative enrichment of H4AC in intronic and exonic regions; and relative depletion of H3AC in intergenic and intronic regions. As in other examples, the *EDLogo* plot is more parsimonious than the wKL-Logo plot.

### Stabilizing $\tilde {\mathbf {r}}$ estimates

The example of *N*-linked glycosylation sites above involves very small (e.g. zero) counts of some amino acids at some sites, and provides an example of both the need to stabilize estimates of $\tilde {r}$ values, and the benefits of our new Empirical Bayes (EB) approach to this.

Before explaining the benefits of our EB approach, we first motivate the need for stabilization, using the central position of this motif as an example. At this position (and, indeed, other positions) the frequency, *p*_*i*_, of amino acid *i* is estimated by counting the number of times, *m*_*i*_, that amino acid *i* occurs in this central position in an observed data set of *m*=5422 sequences. The maximum likelihood estimate (mle) for *p*_*i*_ is *m*_*i*_/*m*. However, *every* observed amino acid at this central position is an Asparagine (*N*), so the mle for *p*_*i*_ is 1 for the *N* amino acid, and is 0 for all other amino acids. These 0 estimates for *p*_*i*_ lead to unreasonably large – indeed, infinite – values for $|\tilde {r}_{i}|$, motivating the need for stabilization.

**Fig. 3:**
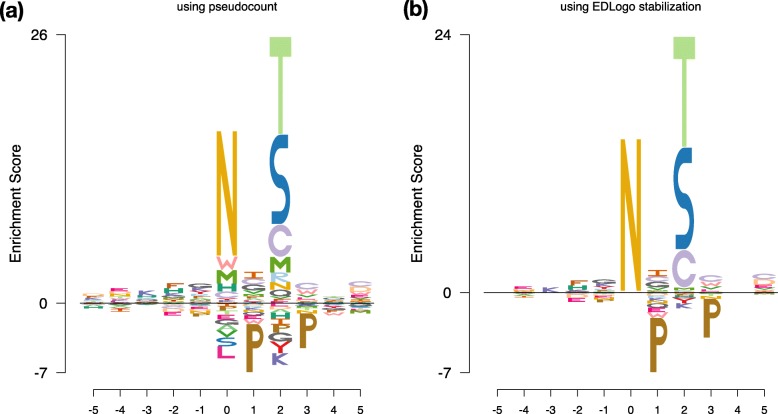
Comparing EDLogo plots from different stabilization methods. The panels show EDLogo plots using (**a**) pseudocount stabilization and (**b**) our new EB stabilization method, for the amino acid motif example from Fig. 2. The EB stabilization produces a more parsimonious plot that better highlights the key enrichments and depletions supported by the data. See text for further discussion

A standard approach to stabilization of estimates of small probabilities is to use pseudocounts. This approach simply adds a pseudocount (small number) to each observed count before computing estimates. For example, using a pseudo-count of 0.5 for each of the 20 amino acids that could occur, *p*_*i*_ is estimated by ${\hat {p}_{i} = (m_{i}+0.5)/(m+10)}$. This avoids zero estimates of probabilities, and an *EDLogo* plot can be constructed from the pseudo-count-based estimates of **p**,**q**. However, we found the resulting plot (Fig. 3a) somewhat unsatisfactory. For example, the approach shows most symbols are either enriched or depleted at the central position, even though the available data can be explained simply by enrichment of *N*. This occurs because, although the estimated *p*_*i*_ are equal for amino acids other than *N*, the estimated background rates *q*_*i*_ vary, and so the estimated $\tilde {r}_{i}$ vary. For example, the pseudocount-based plot shows *W* to be enriched and *L* to be depleted at the central position *even when neither occurs at all in the data*, simply because *L* has a higher background rate.

Our EB stabilization method takes a different approach. Specifically, it directly stabilizes estimates of $\tilde {\mathbf {r}}$, instead of separately stabilizing estimates of **p** and **q** and then taking their log-ratio. Consequently, the method produces estimates of $\tilde {\mathbf {r}}$ that vary no more than is supported by the data, resulting in more parsimonious plots. For example, in this example (Fig. 3b) the plot shows a large *N* alone in the center position, highlighting that the data can be explained purely by strong enrichment of *N*.

In addition, the *EDLogo* strategy of using a median adjustment in (1) to reduce visual clutter can be directly applied to derived quantities such as the position specific scoring matrix (PSSM) commonly used to represent protein binding motifs. Additional file 2: Figure S5 shows logo plots of the PSSM matrix (see Additional file 3: Table S6), before and after median adjustment, of the binding motif of protein *D-isomer specific 2-hydroxyacid dehydrogenase, catalytic domain (IPR006139)* (Motif2,Start = 257, Length = 11) [22, 23]

## Conclusions

We present a new sequence logo plot, the *EDLogo* plot, designed to highlight both enrichment and depletion of elements at each position in a sequence (or other index set). We have also developed statistical methods that can improve these plots by stabilizing enrichment estimates for rare events. We have implemented these methods, as well as standard logo plots, in a flexible R package *Logolas*, which offers many other features: the ability to use strings instead of characters; various customizable styles and color palettes; several methods for scaling stack heights; and ease of integrating logo plots with external graphics like ggplot2 [24].

## Availability and requirements

- Project Name : Logolas
- Software Download page : Github R package (https://github.com/kkdey/Logolas)
- Project Home page : https://kkdey.github.io/Logolas-pages/
- Operating system : Platform independent
- Programming Language : R (≥ 3.4)
- License : GPL (> = 2)
- Any restrictions to use by non-academics: No
- Data : The data used in this paper are reported in Additional file 3 and are also accessible as part of our package Logolas using the *data()* function.
  TFBS example (Fig. 2a, Additional file 2: Figure S1, S6): Additional file 3: Table S1, data(EBF1_disc1)
  N-Glycosylation example (Figs. 2b, 3): Additional file 3: Table S2, data(N_Glycosyl_sequences)
  Mutational Signature example (Fig. 2c, Additional file 2: Figure S3): Additional file 3: Table S3, data(mutation_sig)
  Histone Marks example (Fig. 2d): Additional file 3: Table S4 and S5, data(histone_marks)
  PSSM amino acids example (Additional file 2: Figure S5): Additional file 3: Table S6, data(pssm).

## Additional files

Additional file 1Statistical justification of the Empirical Bayes stabilization of $\tilde {r}$ scores. Also, a proof of the result that median minimizes the sum of absolute deviation and the multiple median scenario for even number of observations - a feature that we exploit in our *EDLogo* scoring. (PDF 219 kb)

Additional file 2**Figure S2.***EDLogo* plots for six different motifs of the EBF1 transcription factor. The PWMS for *known1* and *known2* come from the TRANSFAC database [17]; *known3* from the JASPAR database [16]; *known4* from [18]; *disc1* and *disc2* were discovered by the ENCODE project [14]. Three of the motifs (*known3*, *known4* and *disc1*) show depletion of G and C in the middle of the binding site.**Figure S3.** Comparison of the *EDLogo* plot (a) with *pmsignature* [20] plot (b) for visualizing cancer mutational signatures. Both plots show a cancer mutational signature (signature 12) of from a clustering analysis of somatic mutations by [20]. The *EDLogo* plot highlights the depletion of *G* at the right flanking base more clearly than does the *pmsignature* plot. The use of strings to represent mutations in the center is arguably more intuitive than the *pmsignature* representation.**Figure S4.** Illustration of *EDLogo* for all mutation signatures from Shiraishi et al. *EDLogo* plots for the 27 mutation signature profiles estimated by [20] using data from different cancer types. The heights of the strings in the center of each plot (*C*→*G*, *C*→*T*, etc at position 0 on *x* axis) reflect the relative frequency of each substitution type among somatic mutations contributing to the signature profile, while the heights of the bases at flanking positions on either side reflect the relative frequency of each base at these flanking positions.**Figure S5.** Illustration of median adjustment of a position specific scoring matrix (PSSM). The PSSM shown here is for the binding motif of the protein *D-isomer specific 2-hydroxyacid dehydrogenase, catalytic domain (IPR006139)* (Motif2,Start=257, Length=11). The data has been obtained from the 3PFDB website [22, 23]. The median adjusted PSSM Logo (*bottom panel*) is arguably less cluttered than the non-adjusted version (*top panel*).**Figure S6.** Choice of median. An illustration of how the choice of median value used for centering the $\tilde {r}_{i}$ when the median is an interval (for an even number of characters/classes) can change the *EDLogo* representation of the EBF1-disc1 transcription factor binding site example from Fig. 2 (panel a). In general, choosing the smallest median value favors enrichment of symbols (*top*), whereas choosing the largest median value favors depletion (*bottom*) and choosing the mid-point of the interval treads a common ground between enrichment and depletion (*middle*). As default option in our software and for all the *EDLogo* plots in this paper, we use the smallest median centering. (PDF 2615 kb)

Additional file 3 Supplementary Table. Tables of positional frequency and weight matrices used for creating the different EDLogo plots in the Figures and the Supplementary Figures of the manuscript. (PDF 184 kb)

## Abbreviations

EDLogo: Enrichment depletion logo
KLD: Kullback leibler divergence
PSSM: Position specific scoring matrix
TFBS: Transcription factor binding site
wKL-Logo: weighted Kullback Leibler logo
