# Supplementary material for: A new sequence logo plot to highlight enrichment and depletion
Source: BMC Bioinformatics. 2018 Dec 10;19:473. doi: 10.1186/s12859-018-2489-3 (PMC6288878; doi:10.1186/s12859-018-2489-3)
Supplement: Supplementary file 1 — Statistical justification of the Empirical Bayes stabilization of $\tilde {r}$r~ scores. Also, a proof of the result that median minimizes the sum of absolute deviation and the multiple median scenario for even number of observations - a feature that we exploit in our EDLogo scoring. (PDF 219 kb) [file 12859_2018_2489_MOESM1_ESM.pdf]

Supplementary Methods

Empirical Bayes stabilization of $\tilde{\mathbf{r}}$

Let $\mathbf{p} = (p_1, \ldots, p_M)$ denote the vector of frequencies of M elements $C_1, \cdots, C_M$ (characters or strings) at a particular position i of a logo plot. Assume that $\mathbf{p}$ is to be estimated from N_P observed symbols at that position, and let $\mathbf{x} = (x_1, x_2, \ldots, x_M)$ denote the observed counts of each symbol (so $\sum_{m=1}^{M} x_j = N_P$). Assume a multinomial distribution for $\mathbf{x}$:

$$\mathbf{x} \sim Mult(N_P, \mathbf{p}). \tag{9}$$

Similarly, let $\mathbf{q} = (q_1, \ldots, q_M)$ denote the vector of background frequencies of the M elements, and assume $\mathbf{q}$ is to be estimated from N_Q observed symbols. Let $\mathbf{y} = (y_1, y_2, \ldots, y_M)$ denote the observed counts of each symbol (so $\sum_{m=1}^{M} y_j = N_Q$) and

$$\mathbf{y} \sim Mult(N_Q, \mathbf{q}). \tag{10}$$

Our aim is to estimate $\tilde{r}_j = \log(p_j/q_j)$ from these data $\mathbf{x}, \mathbf{y}$. By assuming N_P and N_Q are large, we can use a Poisson approximation to the Multinomial distributions in Equations (9) and (10):

$$x_j \sim Poi\left(N_P p_j\right) \qquad y_j \sim Poi\left(N_Q q_j\right). \tag{11}$$

Assuming $\mathbf{x}$ and $\mathbf{y}$ are independent, Equation (11) implies

$$x_j | (x_j + y_j) \sim Bin\left(x_j + y_j, \rho_j\right) \quad \text{where} \quad \rho_j := \frac{N_P p_j}{N_P p_j + N_Q q_j} \tag{12}$$

Note that

$$\alpha_j := \log\left(\rho_j/(1-\rho_j)\right) = \log\left(N_P/N_Q\right) + \tilde{r}_j, \tag{13}$$

394 so estimating $\tilde{r}_j$ boils down to estimating α_j.

The maximum likelihood estimate of α_j, given $\mathbf{x}, \mathbf{y}$ and using the likelihood implied by (12), is simply $\log(x_j/y_j)$, which is infinite when x_j or y_j is 0. One way to avoid this infinite estimate is to use Tukey's modification [25]:

$$\hat{\alpha}_j := \begin{cases} \log\left((x_j+0.5)/(y_j+0.5)\right) - 0.5 & \text{if } x_j = 0 \\ \log\left(x_j/y_j\right) & \text{if } x_j = 1, 2, \cdots, N_j - 1 \\ \log\left((x_j+0.5)/(y_j+0.5)\right) + 0.5 & \text{if } x_j = N_j \end{cases} \tag{14}$$

where $N_j = x_j + y_j$. However, this estimate still suffers from high variance when x_j, y_j are 0. To stabilize these estimates we use the Empirical Bayes (EB) approach from Xing and Stephens [26], which in turn is based on methods from [12]. In brief the method combines the estimates (14) with their approximate standard errors [25], given by

$$s_j := \sqrt{V^\star(\hat{\alpha}_j) - 0.5\left\{V_3(\hat{\alpha}_j)\right\}^2 \left\{V_3(\hat{\alpha}_j) - \frac{4}{N_j}\right\}} \tag{15}$$

where

$$V_3(\hat{\alpha}_j) := \frac{N_j+1}{N_j}\left(\frac{1}{x_j+1} + \frac{1}{y_j+1}\right) \qquad V^\star(\hat{\alpha}_j) := V_3(\hat{\alpha}_j)\left\{1 - \frac{2}{N_j} + \frac{V_3(\hat{\alpha}_j)}{2}\right\}. \tag{16}$$

The EB approach from [12], implemented in the **ashr** package, takes as input any set of estimates and corresponding standard errors, and outputs shrunken (stabilized) estimates. We apply this approach to the estimates (14) and their standard errors (15) to obtain stabilized estimates, $\alpha_j^\star$, for α_j. (Note that while [12] focuses on the case where the prior distribution is unimodal about 0, the software has the option to estimate the location of the mode, and we use this option here.)

Finally, using (13), we obtain

$$\tilde{\hat{r}}_j = \alpha_j^\star - \log\left(N_P/N_Q\right). \tag{17}$$

Median minimizes the sum of absolute deviations

Say we have n points $x_1, x_2, \cdots, x_n$. We order them $x_{(1)} < x_{(2)} < \cdots x_{(n)}$. Suppose we want to find the a that minimizes

$$\underset{a}{argmin} \sum_{i=1}^{n} |x_i - a|$$

We show that when n is odd, say $n = 2m + 1$, then the a that minimizes the above quantity is $a^\star = x_{(m+1)}$, which is the median point. If n is even, say $n = 2m$, then the minimizing $a^\star$ could be any value between $x_{(m)}$ and $x_{(m+1)}$, the interval between the two middle points.

The subgradient of $\sum_{i=1}^{n} |x_i - a|$ with respect to a is given by

$$\delta(a) = \sum_{i=1}^{n} sgn(x_i - a) \tag{18}$$

The minimizing value of a is the one for which $\delta(a)$ is equal to 0.

When $n = 2m + 1$, $\delta(a)$ equals 0 when a is equal to the middlemost x_i value, namely $x_{(m+1)}$, as $sgn(x_{(i)} - x_{(m+1)}) = -1$ for the m values such that $i \leq m$ and $sgn(x_{(i)} - x_{(m+1)}) = 1$ for the m values from $i = m + 2, \cdots, 2m + 1$, and $sgn(x_{(m+1)} - x_{(m+1)}) = 0$

$$\delta(a) = \sum_{i=1}^{m} sgn(x_{(i)} - a) + sgn(x_{(m+1)} - a) \sum_{i=m+2}^{2m+1} sgn(x_{(i)} - a) = -m + 0 + m = 0$$

When $n = 2m$, $\delta(a)$ equals to 0 when a is any value between $x_{(m)}$ and $x_{(m+1)}$. because when $x_{(m)} < a < x_{(m+1)}$, for the m values $i \leq m$ we have $sgn(x_{(i)} - a) = -1$ and for the remaining m values $i = m + 1, \cdots, 2m$ we have $sgn(x_{(i)} - a) = +1$, so

$$\delta(a) = \sum_{i=1}^{m} sgn(x_{(i)} - a) + \sum_{i=m+1}^{2m} sgn(x_{(i)} - a) = -m + m = 0$$

The above analysis only shows that the median is a local optima. That it is a minima is easily seen by choosing a to be outside the range of the x_i's for which the sum of absolute deviations will be greater than any a inside the range. The fact that this local minima is global follows from the convexity of the function $\sum_{i=1}^{n} |a - x_i|$ with respect to a ($f(y) = |y|$ is convex and sum of convec functions is convex).
