# Supplementary material for: A new sequence logo plot to highlight enrichment and depletion
Source: BMC Bioinformatics. 2018 Dec 10;19:473. doi: 10.1186/s12859-018-2489-3 (PMC6288878; doi:10.1186/s12859-018-2489-3)
Supplement: Supplementary file 2 — Figure S2.EDLogo plots for six different motifs of the EBF1 transcription factor. The PWMS for known1 and known2 come from the TRANSFAC database [17]; known3 from the JASPAR database [16]; known4 from [18]; disc1 and disc2 were discovered by the ENCODE project [14]. Three of the motifs (known3, known4 and disc1) show depletion of G and C in the middle of the binding site. Figure S3. Comparison of the EDLogo plot (a) with pmsignature [20] plot (b) for visualizing cancer mutational signatures. Both plots show a cancer mutational signature (signature 12) of from a clustering analysis of somatic mutations by [20]. The EDLogo plot highlights the depletion of G at the right flanking base more clearly than does the pmsignature plot. The use of strings to represent mutations in the center is arguably more intuitive than the pmsignature representation. Figure S4. Illustration of EDLogo for all mutation signatures from Shiraishi et al. EDLogo plots for the 27 mutation signature profiles estimated by [20] using data from different cancer types. The heights of the strings in the center of each plot (C→G, C→T, etc at position 0 on x axis) reflect the relative frequency of each substitution type among somatic mutations contributing to the signature profile, while the heights of the bases at flanking positions on either side reflect the relative frequency of each base at these flanking positions. Figure S5. Illustration of median adjustment of a position specific scoring matrix (PSSM). The PSSM shown here is for the binding motif of the protein D-isomer specific 2-hydroxyacid dehydrogenase, catalytic domain (IPR006139) (Motif2,Start=257, Length=11). The data has been obtained from the 3PFDB website [22, 23]. The median adjusted PSSM Logo (bottom panel) is arguably less cluttered than the non-adjusted version (top panel). Figure S6. Choice of median. An illustration of how the choice of median value used for centering the [file 12859_2018_2489_MOESM2_ESM.pdf]

421 # Supplementary Figures

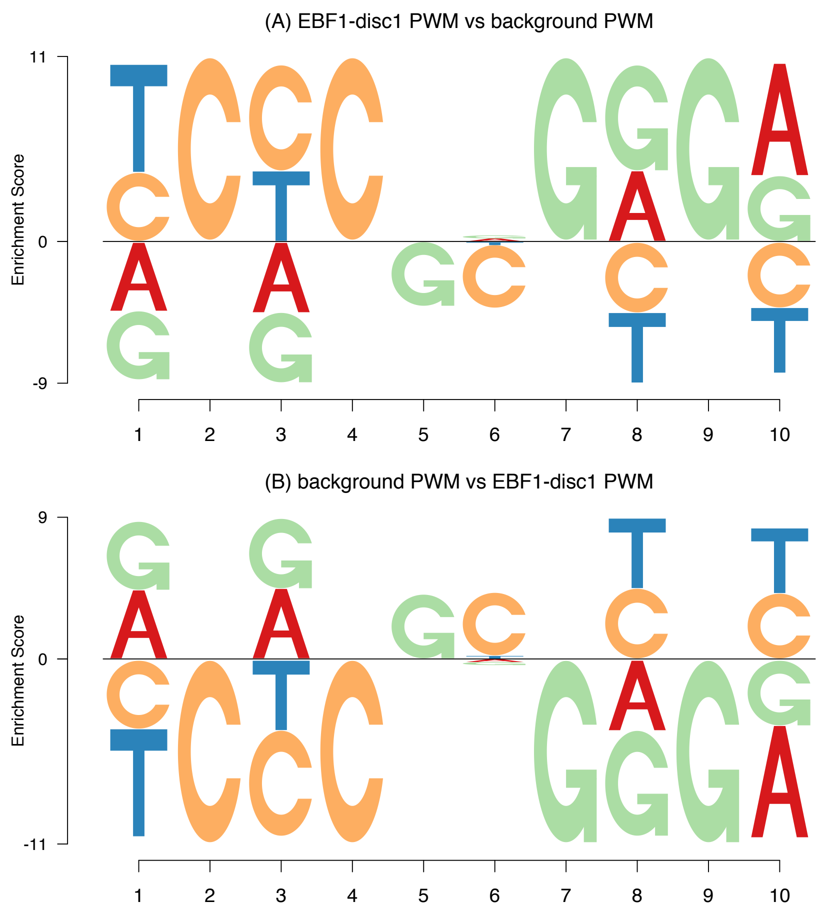

Figure S1 Illustration of "mirror property" of *EDLogo*. *Panel (a)*: *EDLogo* plot of the position weight matrix (PWM) of the primary discovered motif *disc1* from [14] of the EBF1 transcription factor against uniform background, with the conventional median adjustment. *Panel (b)*:*EDLogo* plot of a uniform PWM against the PWM of EBF1 as background. That is, panels (a) and (b) are comparing the same two PWMs, but differ in which one they treat as the "background". The *EDLogo* plot obeys the mirror property, in that (b) is a mirror image of (a) (modulo the orientation of the symbols, which are translated and not reflected).

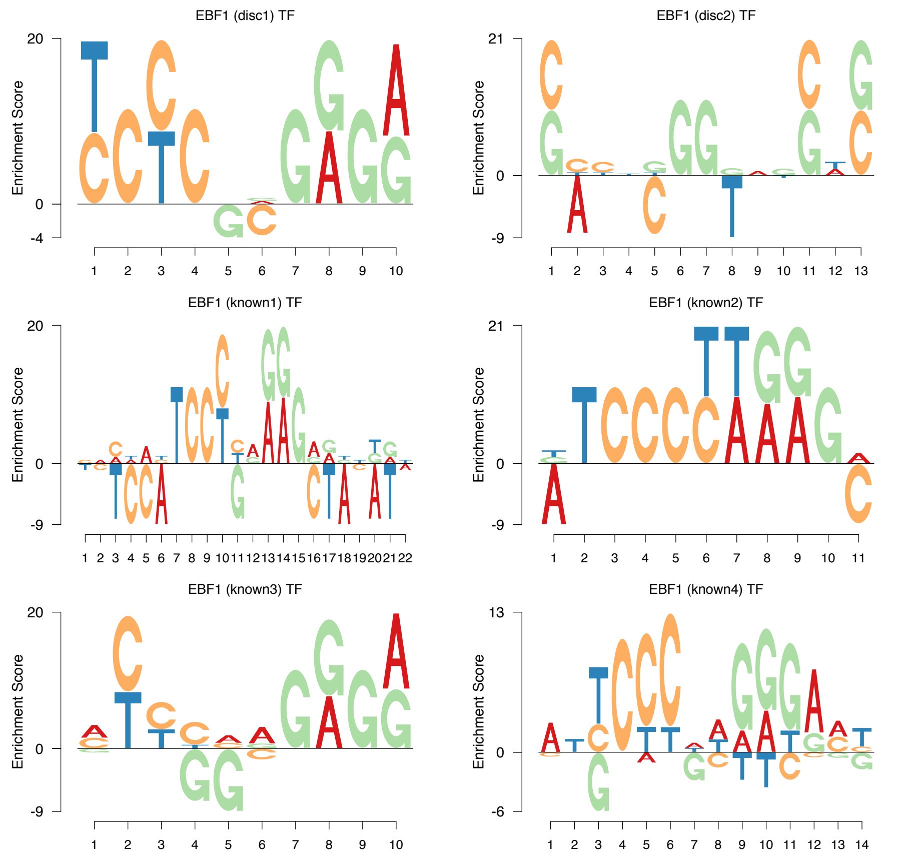

Figure S2 ***EDLogo*** **plots for six different motifs of the EBF1 transcription factor.** The PWMS for *known1* and *known2* come from the TRANSFAC database [17]; *known3* from the JASPAR database [16]; *known4* from [18]; *disc1* and *disc2* were discovered by the ENCODE project [14]. Three of the motifs (*known3*, *known4* and *disc1*) show depletion of G and C in the middle of the binding site.

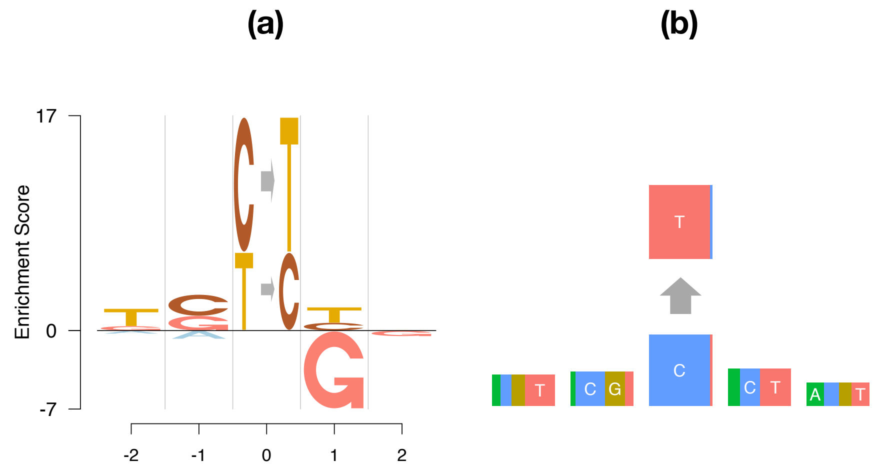

Figure S3 Comparison of the *EDLogo* plot (a) with *pmsignature* [20] plot (b) for visualizing cancer mutational signatures. Both plots show a cancer mutational signature (signature 12) of from a clustering analysis of somatic mutations by [20]. The *EDLogo* plot highlights the depletion of G at the right flanking base more clearly than does the *pmsignature* plot. The use of strings to represent mutations in the center is arguably more intuitive than the *pmsignature* representation.

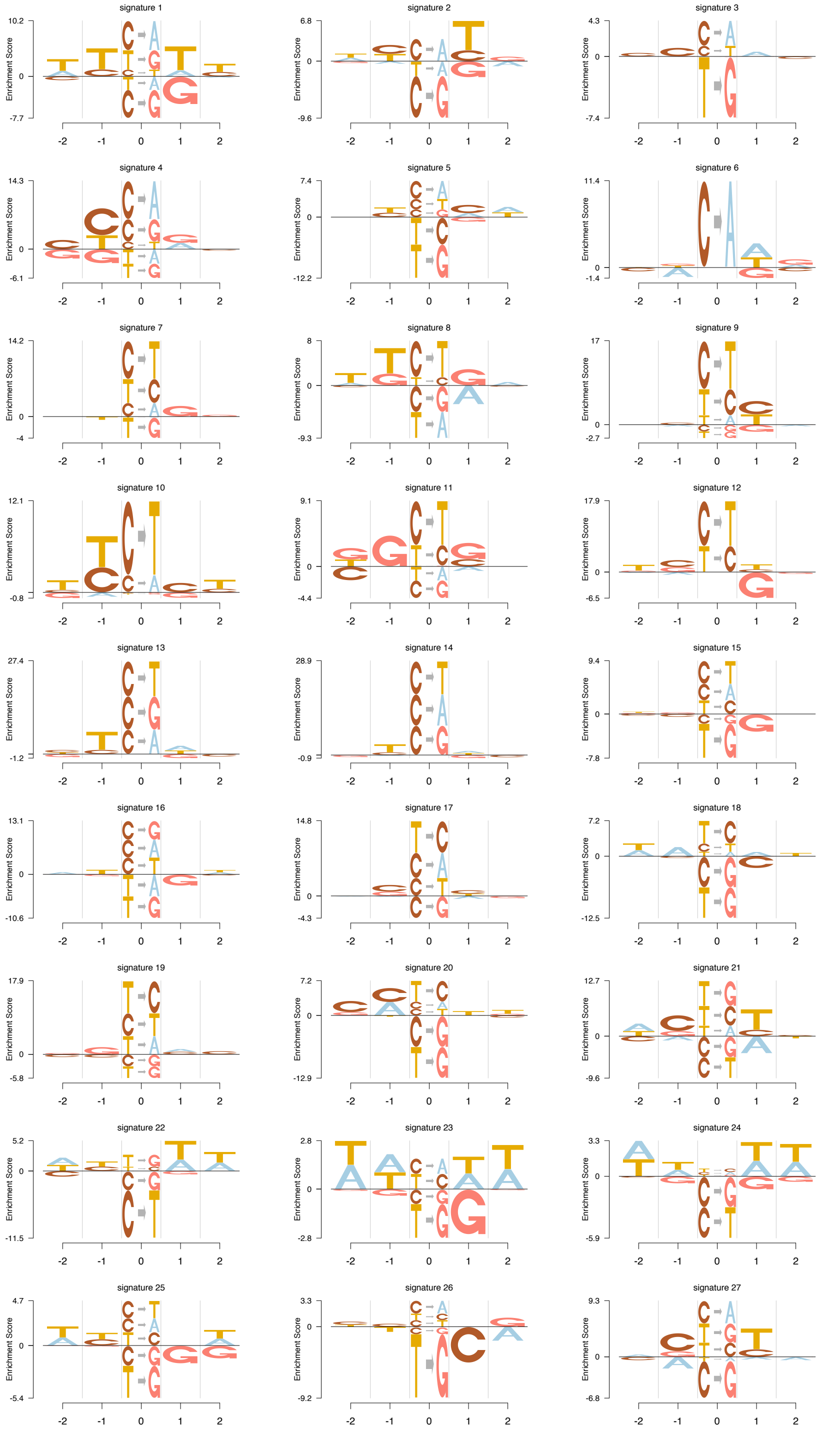

Figure S4 Illustration of *EDLogo* for all mutation signatures from Shiraishi et al: *EDLogo* plots for the 27 mutation signature profiles estimated by [20] using data from different cancer types. The heights of the strings in the center of each plot ($C \to G$, $C \to T$, etc at position 0 on x axis) reflect the relative frequency of each substitution type among somatic mutations contributing to the signature profile, while the heights of the bases at flanking positions on either side reflect the relative frequency of each base at these flanking positions.

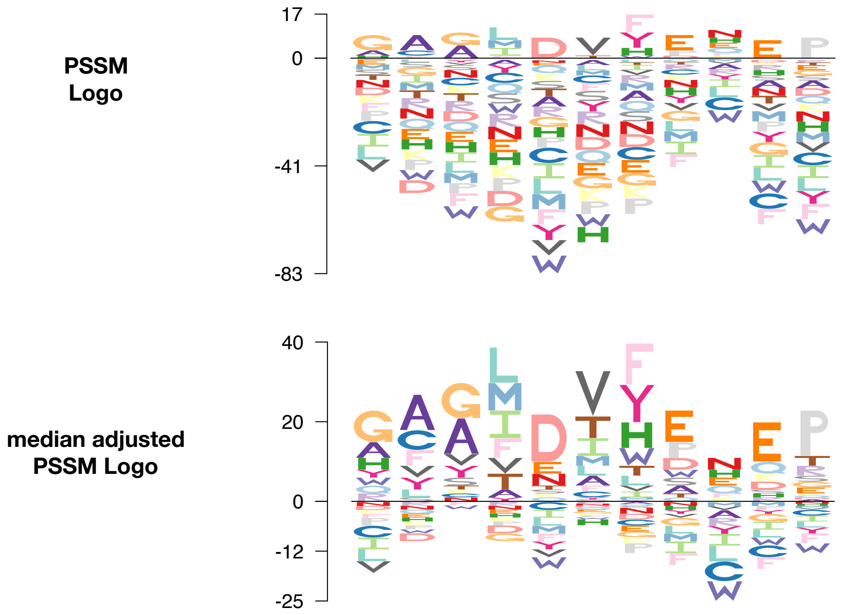

Figure S5 Illustration of median adjustment of a position specific scoring matrix (PSSM). The PSSM shown here is for the binding motif of the protein *D-isomer specific 2-hydroxyacid dehydrogenase, catalytic domain (IPR006139)* (Motif2,Start=257, Length=11). The data has been obtained from the 3PFDB website [22, 23]. The median adjusted PSSM Logo (*bottom panel*) is arguably less cluttered than the non-adjusted version (*top panel*).

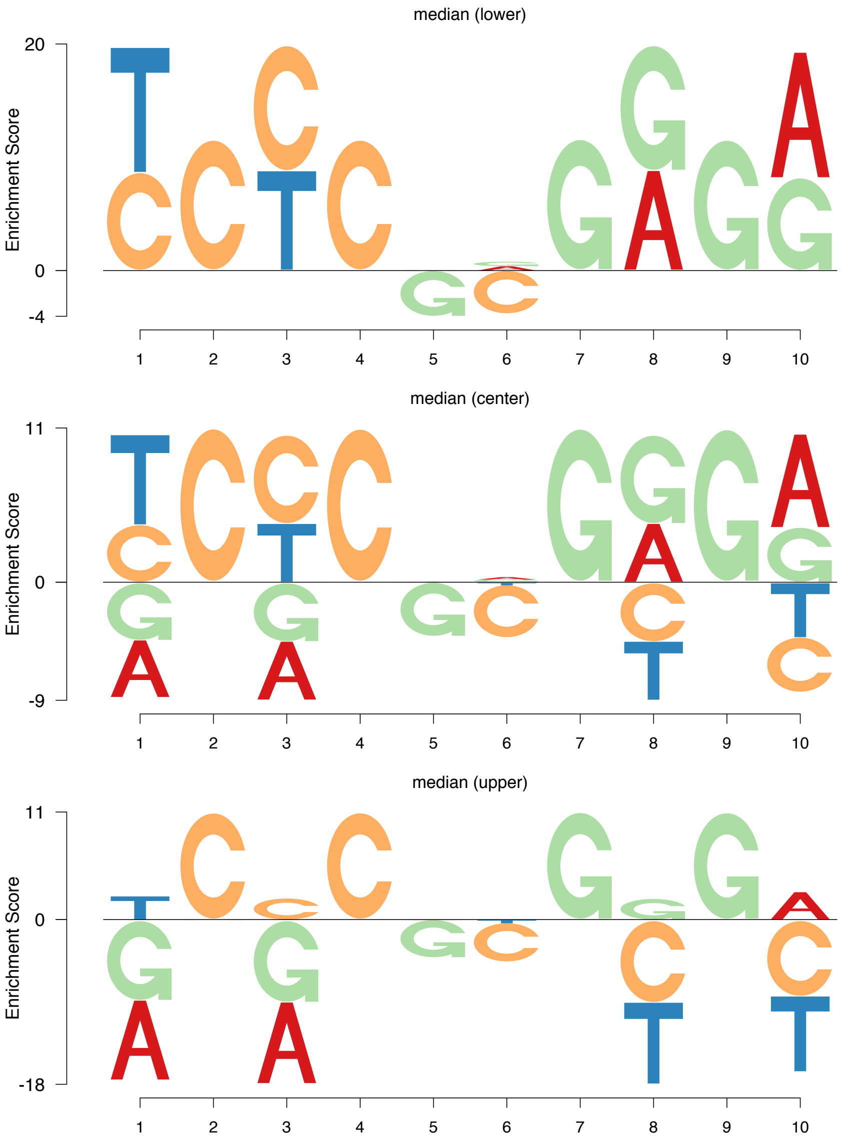

Figure S6 Choice of median. An illustration of how the choice of median value used for centering the $\tilde{r}_i$ when the median is an interval (for an even number of characters/classes) can change the *EDLogo* representation of the EBF1-disc1 transcription factor binding site example from Figure 2 (panel a). In general, choosing the smallest median value favors enrichment of symbols (*top*), whereas choosing the largest median value favors depletion (*bottom*) and choosing the mid-point of the interval treads a common ground between enrichment and depletion (*middle*). As default option in our software and for all the *EDLogo* plots in this paper, we use the smallest median centering.
