# Supplementary material for: A new sequence logo plot to highlight enrichment and depletion
Source: BMC Bioinformatics. 2018 Dec 10;19:473. doi: 10.1186/s12859-018-2489-3 (PMC6288878; doi:10.1186/s12859-018-2489-3)
Supplement: Supplementary file 3 — Supplementary Table. Tables of positional frequency and weight matrices used for creating the different EDLogo plots in the Figures and the Supplementary Figures of the manuscript. (PDF 184 kb) [file 12859_2018_2489_MOESM3_ESM.pdf]

Supplementary Tables

Table S1 The position weight matrix (PWM) $\mathbf{p}$ for the EBF1-disc1 transcription factor. The columns represent the base positions and the rows are bases, with each entry of the matrix representing the relative frequency of a base in the position represented by the column. The PWM data has been fetched from http://compbio.mit.edu/encode-motifs/ [14]. The background probabilities $\mathbf{q}$ were considered equal (all 0.25) for all positions.

	1	2	3	4	5	6	7	8	9	10
A	0	0	0	0	0.298	0.336	0	0.177	0	0.885
C	0.153	1	0.826	1	0.357	0.021	0	0	0	0
G	0	0	0	0	0.021	0.375	1	0.823	1	0.115
T	0.847	0	0.174	0	0.324	0.268	0	0	0	0

Table S2 Position Weight Matrix for N-linked glycosylation motifs, comprising of the N-linked site and the neighboring bases (5 before and 5 after the N) surrounding it. The data was downloaded from the UniprotKB protein database [19]. Background probabilities were computed by the relative proportion of the median counts of each amino acid over the 11 positions. The effective count for the background probabilities was also taken to be 5422, the number of sequences in the original data.

	-5	-4	-3	-2	-1	0	1	2	3	4	5
A	312	311	348	365	385	0	402	2	366	348	355
C	142	147	148	125	145	0	225	14	244	149	212
D	266	272	225	177	196	0	201	1	268	234	320
E	320	393	323	249	280	0	252	3	335	336	376
F	266	226	231	330	267	0	268	2	260	203	229
G	341	325	358	389	450	0	508	1	337	305	310
H	119	137	156	188	136	0	133	0	147	149	130
I	283	242	279	277	272	0	440	1	293	310	311
K	237	228	283	243	257	0	179	0	206	205	204
L	533	594	537	544	566	0	581	4	643	524	484
M	93	94	92	105	100	0	109	2	87	97	73
N	216	257	209	230	181	5422	187	3	181	225	225
P	325	325	312	322	256	0	10	1	28	344	317
Q	231	226	260	228	242	0	180	3	241	265	210
R	260	234	267	224	274	0	206	4	242	260	229
S	443	424	414	427	395	0	448	2365	432	434	388
T	349	356	301	317	284	0	318	3012	277	369	287
V	390	348	379	370	385	0	494	4	493	401	439
W	91	101	96	82	97	0	62	0	135	75	110
Y	205	182	204	230	254	0	219	0	207	189	213

Table S3 The position weight matrix (PWM) $\mathbf{p}$ for mutation signature profile 12 in [20]. The background was chosen to be of equal probability (1/4 for the flanking positions, and 1/6 for the mutation at the center, as there are 6 possibilities).

-2 left flank	A: 0.143, C: 0.169, G: 0.219, T: 0.469
-1 left flank	A: 0.085, C: 0.464, G: 0.317, T: 0.133
mutation	$C \to A$: 7e-10, $C \to G$: 0, $C \to T$: 0.965, $T \to A$: 0 $T \to C$: 0.035, $T \to G$: 0
+1 right flank	A: 0.197, C: 0.313, G: 0.002, T: 0.487
+2 right flank	A: 0.277, C: 0.245, G: 0.193, T: 0.284

Table S4 The observed distribution of histone marks in different regions of the genome for the lymphoblastoid cell line GM06990, as presented in Table S2 (upper) of [10].

	Intergenic	Intron	Exon	Gene start	Gene end
H3K4ME1	326	296	81	245	71
H3K4ME2	258	228	55	273	90
H3K4ME3	145	121	29	253	85
H3AC	60	52	23	180	53
H4AC	150	191	63	178	63

Table S5 The background distribution of histone marks in different regions of the genome, as presented in Table S2 (lower) of [10].

	Intergenic	Intron	Exon	Gene start	Gene end
H3K4ME1	542	218	118	108	33
H3K4ME2	480	204	107	87	26
H3K4ME3	346	131	71	66	20
H3AC	199	72	41	43	13
H4AC	368	101	67	80	30

Table S6 Position Specific Scoring Matrix (PSSM) data for the binding motif of the protein *D-isomer specific 2-hydroxyacid dehydrogenase, catalytic domain (IPR006139)* (Motif2,Start=257, Length=11) [22, 23].

	257	258	259	260	261	262	263	264	265	266	267
A	2	6	5	-2	-4	-2	-4	0	-2	-3	-3
R	0	-4	-4	-5	-4	-5	-3	-2	-2	-2	-1
N	-3	-4	-3	-5	-1	-5	-5	-3	3	-3	-4
D	-3	-5	-4	-6	8	-5	-5	1	0	-1	-3
C	-5	2	-3	-4	-6	-3	-5	-2	-5	-6	-5
Q	0	-4	-4	-4	-3	-5	-4	-1	2	0	-3
E	-2	-4	-4	-5	-1	-5	-5	6	2	7	-2
G	6	-3	5	-6	-4	-5	-5	-4	0	-5	-2
H	1	-4	-4	-5	-4	-6	3	-3	2	-2	-4
I	-5	-3	-4	3	-6	0	-2	-5	-3	-5	-5
L	-5	-1	-4	5	-6	-2	-1	-4	-4	-5	-5
K	-3	-4	-3	-5	-3	-5	-5	-2	1	-1	-3
M	-2	-3	-4	3	-6	-2	-3	-4	-1	-4	-4
F	-3	1	-5	1	-6	-3	7	-5	1	-6	-6
P	-4	-4	-4	-5	-4	-5	-6	2	0	-4	8
S	-2	-2	-2	-4	-3	-4	-4	0	0	-2	-2
T	-2	-3	-3	0	-3	1	-1	-1	0	-3	-1
W	0	-4	-5	-4	-7	-5	1	0	-5	-5	-6
Y	0	0	-1	-3	-6	-4	6	-3	-2	-4	-5
V	-5	0	-1	0	-6	7	-2	-3	-1	-3	-4
